# Model-based translation of the PKPD-relationship for linezolid and vancomycin on methicillin-resistant *Staphylococcus aureus*: from *in vitro* time–kill experiments to a mouse pneumonia model

**DOI:** 10.1093/jac/dkaf140

**Published:** 2025-05-09

**Authors:** Diego Vera-Yunca, Carina Matias, Carina Vingsbo Lundberg, Lena E Friberg

**Affiliations:** Department of Pharmacy, Uppsala University, Uppsala, Sweden; Bacteria, Parasites & Fungi, Statens Serum Institut, Copenhagen, Denmark; Bacteria, Parasites & Fungi, Statens Serum Institut, Copenhagen, Denmark; Department of Pharmacy, Uppsala University, Uppsala, Sweden

## Abstract

**Objectives:**

MRSA is one of the main pathogens that cause nosocomial pneumonia. Based on longitudinal *in vitro* and *in vivo* data, a pharmacokinetic–pharmacodynamic (PKPD) model was built to quantify the effect of two control antibiotics (LZD and VAN) for Gram-positive bacteria in a standardized mouse pneumonia model

**Methods:**

The PKPD model was developed for data generated on the MRSA strain 160 079 in static *in vitro* time–kill experiments and thereafter adjusted to fit data from lungs of neutropenic mice administered with single or multiple doses of LZD (0.5–40 mg/kg) or VAN (1–40 mg/kg). Simulations with human PK were run to predict antibacterial response in patients.

**Results:**

Bacterial regrowth observed *in vitro* when exposed to VAN concentrations was described by an adaptive resistance model. The selected MRSA isolate showed good virulence in the mouse pneumonia model. Bacterial load in lungs decreased up to 2-log with respect to control mice after LZD and VAN treatment. A 70%–75% lower killing rate was estimated for the *in vivo* data when compared with *in vitro*. Simulations displayed bacterial stasis at 24 h for patients infected with bacteria with MICs below the clinical breakpoint for both drugs after administering standard-of-care dosing regimens.

**Conclusions:**

A translational workflow allowed us to build a PKPD model with both *in vitro* and *in vivo* data that characterized bacterial dynamics following LZD and VAN exposure, showing that this approach can inform the development of antibiotics. We also showcased the first successful use of the standardized mouse pneumonia model for Gram-positive bacteria.

## Introduction

Nosocomial pneumonia (either hospital-acquired or ventilation-associated) is one of the most common infectious diseases in hospitalized patients, second only to urinary tract infections.^1,2^ One of the main pathogens that cause pneumonia in the hospital setting is MRSA.^3,4^ The standard-of-care antibiotic for treating MRSA pneumonia is either VAN or LZD.^5,6^ In order to further explore new treatments for pneumonia, standardized animal models are of value to better leverage experimental results by reducing intra- and inter-laboratory experimental variability and increase animal model translational performance. Recently, as part of the COMBINE project (https://amr-accelerator.eu/project/combine/), a standardized mouse pneumonia model for Gram-negative infections was developed,^7^ aimed at harmonizing the experimental setup and facilitate comparisons between labs and compounds. However, the model has not been tested for Gram-positive bacteria (specifically MRSA) or been explored for its *in vitro*–*in vivo* translational capabilities.

To evaluate the effectiveness of antibiotics, pharmacokinetics (PK) and bacterial susceptibility, represented as the MIC, are combined into a single parameter known as the PK/pharmacodynamic (PD) index. This index is used to determine the optimal antibiotic dosage in clinical practice. For both VAN^8–10^ and LZD,^8,11,12^ the PK/PD index is the ratio of the area under the curve (AUC) and the MIC (AUC/MIC). However, the ‘best’ PK/PD index and its target value can vary depending on the conditions—not only among bacterial species and strains but also across host species and patient populations. This uncertainty makes translating preclinical findings into clinical applications more challenging,^13^ as discussed earlier.

A more robust approach to the analysis of preclinical data for translational purposes is the usage of mathematical PKPD models.^13,14^ These allow for considering the differences in PK, bacterial growth and antimicrobial effect (and resistance to the compound if it arises) across bacterial strains, animal models and humans. The semi-mechanistic PKPD model is first developed with rich *in vitro* data from ‘static’ (exposing the bacteria to constant drug concentrations) and/or ‘dynamic’ (complex experimental systems that mimic drug PK) bacterial time–kill experiments. Future animal experiments can be informed based on the predictions of these *in vitro* mathematical PKPD models. Then, the *in vitro* model parameters can be refined by including these new *in vivo* observations. By coupling this modelling framework with human PK models, predictions of the change in bacterial load over time in patients can be obtained under different simulated dosing regimens. This model-based translation workflow has been successfully applied to different antibiotic classes.^15–20^ Furthermore, this translational approach has been used for Gram-negative bacteria in pneumonia by considering *in vivo* lung bacterial burden^21^ but has not, as far as we are aware, been applied to Gram-positive bacteria in pneumonia. Therefore, it would be useful to have a PKPD modelling framework translating from *in vitro* to *in vivo* that facilitates the development of new anti-infective against MRSA. The PKPD model could be further adapted to, for example suggest human efficacious dose from preclinical results and predict human clinical studies.

Therefore, the aims of this work were (1) to evaluate whether the standardized mouse pneumonia infection model^7^ is suitable for Gram-positive bacteria experiments, (2) to build a semi-mechanistic PKPD model for VAN and LZD in MRSA that described both *in vitro* and *in vivo* data and (3) to link the aforementioned PKPD model with human PK models for both drugs to described the expected bacterial burden in patients.

## Materials and methods

### Ethics

Mouse studies were carried out at Statens Serum Institut in Copenhagen, Denmark. All animal experiments were approved by the National Animal Experiments Inspectorate (licence 2021-15-0201-01055) and are in adherence to the European Directive 2010/63/EEC.

### Strains

A respiratory tract clinical isolate recovered from tracheal secretions, MRSA 160079 (LZD MIC = 2 mg/L, VAN MIC = 1 mg/L), was selected after confirming the *in vivo* growth capabilities in preliminary virulence studies in the mouse pneumonia model.

### *In vitro* time–kill curves

The drug-mediated bactericidal effect was analysed by performing time–kill experiments. The bacterial inoculum was prepared to a final concentration of 10^5^ cfu/mL in test plates. Either linezolid or VAN was added as a single agent to achieve static concentrations ranging from 0.5 to 16×MIC (LZD) or from 0.25 to 4×MIC (VAN). Test plates were incubated at ±35°C with shaking for 26 h and samples were taken at timepoints 0, 1, 2, 4, 6, 8, 24 and 26 h, for cfu determination. The limit of detection was set to 1 log_10_ cfu per mL (cfu/mL). Initially, three time–kill replicates were planned for each compound. In the end, four replicates were performed for LZD to investigate reproducibility, while one of the three VAN replicates was deemed not suitable for the analysis and removed from the study.

### *In vivo* treatment studies

*In vivo* experiments were performed using a standardized protocol for a neutropenic mouse lung infection model^7^ that has been developed as one of the main aims of the COMBINE project (https://amr-accelerator.eu/project/combine/). More details about neutropenia induction and infection procedures are provided in Supplementary material S1 (available as Supplementary data at *JAC* Online).

Drug efficacy studies were carried out using the mouse pneumonia model described above (Supplementary material S2). A single subcutaneous dose of either LZD or VAN (40 mg/kg, 2 h after bacterial inoculation) was evaluated for bactericidal effect. In this study, a total of 65 mice, divided into 13 groups (*n* = 5), were used. One group was sacrificed 2 h after bacterial inoculation to determine bacterial load at the start of treatment, and 12 groups were treated with either a saline solution (vehicle), LZD or VAN. Five mice were euthanized from each group at 4, 8, 12 or 26 h (or when the clinical score reached the ethical endpoint). Measurements were expressed as log_10_ cfu per two lungs (total lung).

Based on the first experiment’s results, a second experiment was run with the same mouse model but with repeated subcutaneous dosing. A total of 14 groups comprising 70 mice in total were included in this experiment. LZD doses ranged from 0.5 to 30 mg/kg q4h, while VAN doses ranged between 1 and 30 mg/kg q8h. Most mice were set to be sacrificed at 26 h (or earlier if reaching the humane endpoint). In addition, for LZD 8 mg/kg q8h and VAN 10 mg/kg q4h, there were also groups euthanized at either 8 or 12 h, resulting in a longitudinal profile of the bacterial killing.

### PKPD modelling

#### PKPD model for *in vitro* data

**Figure 1.**
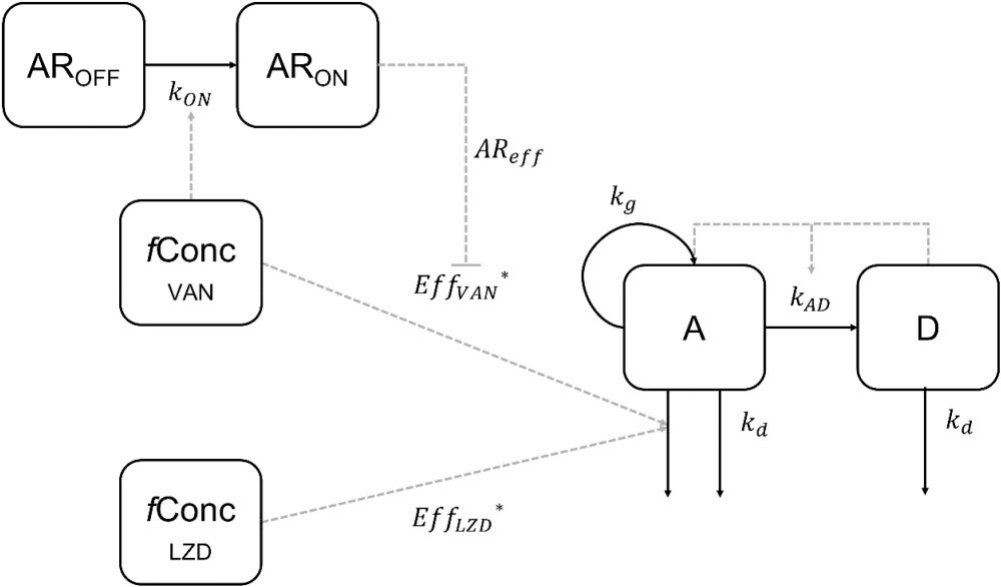
Schematic representation of the final PKPD model. AR_OFF_, no adaptive resistance to VAN; AR_ON_, adaptive resistance to VAN; *f*Conc_VAN_ and *f*Conc_LZD_, free concentration from either *in vitro* (static concentration) or from *in vivo* (mouse plasma compartment) experiments for VAN and LZD, respectively. A, active bacteria; D, dormant bacteria; *k*_ON_, second-order rate constant for development of VAN adaptive resistance; Eff_VAN_ and Eff_LZD_, VAN and LZD drug effects, respectively. AR_eff_, adaptive resistance effect on VAN drug effect; *k*_g_, bacterial growth rate constant; *k*_d_, bacterial natural death rate constant; *k*_AD_, transfer rate constant from A to D; *, drug concentration that exerts its effect *in vivo* comes from an effect compartment (not shown in the figure).

A two-state bacterial growth model was implemented,^22^ consisting of a growing (at a rate defined as *k*_g_) drug-susceptible, active state (*A*) and a non-growing, non-susceptible dormant state (*D*), as shown in Figure 1. All model structure equations are shown in supplementary material S3. All bacteria were in the *A* state at time = 0, and could transit to the *D* state when the bacterial density increased. A fixed natural death rate of 0.179 h^−1^ accounted for the non-drug-induced bacterial death.^22^ Different drug effect models, such as linear, exponential, *E*_max_ or sigmoidal *E*_max_ were tested during the data analysis.

The observed bacterial regrowth was assessed by evaluating different bacterial drug resistance mechanisms. A model structure that included a bacterial adaptive resistance mechanism^15^ was one of the bacterial regrowth models tested during the analysis. Exposure of bacteria to drug concentrations increased the degree of bacterial resistance over time. Other mechanisms, such as the two-subpopulation model^23^ (two pre-existing bacterial subpopulations at the start of the experiment, one being more susceptible and the other one more resistant to the drug), were also explored.

#### PKPD model adjustments for *in vivo* data

Bacterial growth dynamics in control mice (that received a saline solution) were different when compared with *in vitro*. Therefore, growth-related model parameters were re-estimated by including only virulence data from untreated mice. The resulting growth parameter estimates were fixed when assessing the *in vivo* drug effect. PK models after an intravenous administration were adapted from the literature^24,25^ and it was assumed the subcutaneous absorption was very fast and complete (Supplementary material S4). These models were used to predict the unbound drug concentration–time profiles driving the PKPD model’s drug effect. Mouse treatment studies presented sparser data than *in vitro* PKPD experiments, as studies were terminal and only a single-timepoint could be obtained from each mouse. This resulted that the parameters from the semi-mechanistic PKPD model developed with *in vitro* data could not be identified based on the *in vivo* data alone. Therefore, all relevant drug effect parameters were re-estimated one by one. The final PKPD model for *in vivo* data was obtained after estimating *in vivo* growth parameters and the statistically different (compared with *in vitro)* drug effect parameters that were selected in the previous step.

### Simulations of bacterial load over time with human PK

The final PKPD model refined with *in vivo* data was coupled to human PK models of LZD^26^ and VAN^27^ to explore the predicted bacterial burden over time for standard dosing regimens in the clinic: 600 mg q12h as an intravenous infusion of an hour (LZD), and 15 mg/kg q12h as an intravenous infusion of either 1 h or with a rate of 600 mg/h, whichever is the slowest (VAN). Patients’ weights were set to 70 kg, and the rest of the covariates were set to the PK models’ reference values: creatinine clearance (80 mL/min) and weight (69.5 kg). Unbound fraction values were set to 0.88^26^ and 0.45^28^ for LZD and VAN, respectively. MIC effect on *EC_50_* was considered as shown in supplementary material S3.5, equation 11. MIC values around the clinical breakpoints for LZD and VAN (defined by EUCAST^29^) were simulated. Model simulations accounted for inter-individual variability of human PK models, as well as the residual variability and parameter uncertainty of the final PKPD model.

### Model selection and evaluation

The selection of the different PKPD model candidates for both *in vitro* and *in vivo* data was carried out based on several criteria. A decrease in the objective function value (OFV) of 3.84 points (for a *P* value of 0.05) was considered as statistically significant. The precision of the parameter estimates, as evaluated based on the relative standard errors (RSEs), was also considered. Goodness-of-fit plots were also assessed. Parameter uncertainty was further explored with the sampling importance resampling (SIR) tool.^30^ Model ability to reproduce the median observed data was evaluated by using the Visual Predictive Check (VPC)^31^ tool and by running model simulations with residual variability and parameter uncertainty.

### Software list

R^32^ was used for dataset preparation and model output processing, including plots. NONMEM v7.5.0 (Icon PLC, Dublin, Ireland) was used for model fitting, and together with PsN v5.3.0,^33^ running the SIR and VPC tools for model evaluation. Model management was carried out in Pirana 2.9.6.^34^ Simulations of preclinical and clinical scenarios were run in mrgsolve,^35^ an R package.

## Results

### PKPD model for *in vitro* time–kill curve data

**Figure 2.**
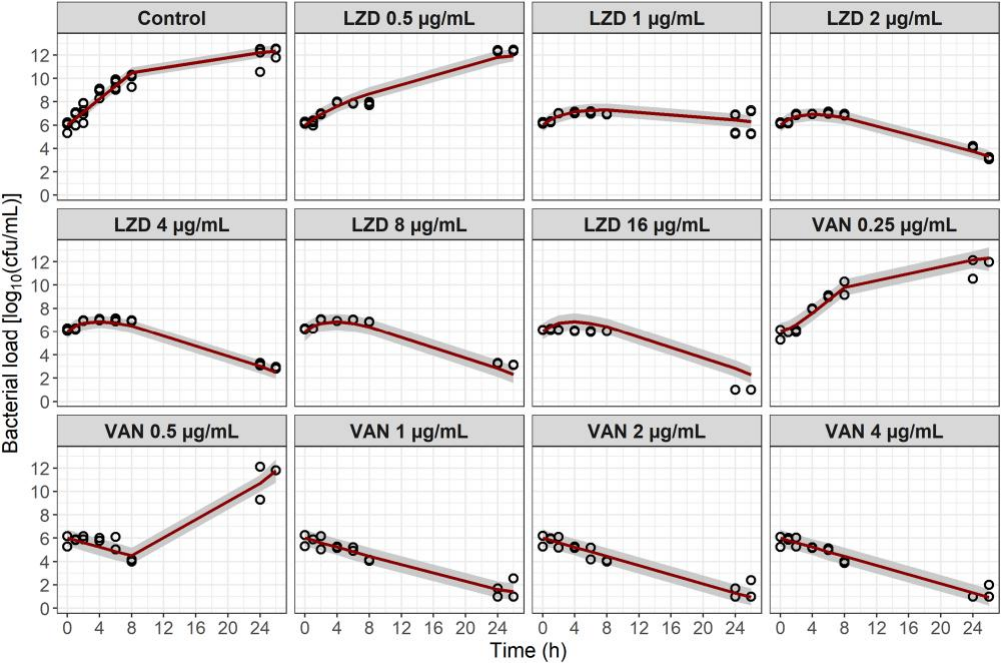
Visual predictive checks of the final PKPD model for *in vitro* data. A total of 1000 studies with the same design as the performed experiments were simulated. Open circles, observed bacterial load. Solid lines, median simulated bacterial load. Grey lines, 95% confidence interval for the simulated median.

The MRSA 160 079 strain showed concentration-dependent bacterial killing when exposed to both LZD and VAN. The final PKPD model structure (Figure 1) successfully described bacterial dynamics over time with and without the presence of LZD or VAN in all experimental scenarios (Figure 2). After testing different drug effect models, a sigmoidal *E*_max_ model was chosen for both drugs. Bacterial regrowth after the early killing phase was observed for VAN at half the MIC (0.5 mg/L). This effect was captured by the adaptive resistance mechanism included in the model. A delay in LZD effect improved the model significantly (OFV drop of 142.3 points). A similar delay did not improve the fit of the VAN data. Since there was no washout period during these experiments, it was impossible to estimate the transfer rate from state D to state A, nor the adaptive resistance rate that controls the transfer from AR_on_ back to AR_off_ (Figure 1, supplementary material S3). The estimation of the Hill coefficient of the VAN drug effect was imprecise and resulted in a high value. Therefore, it was fixed to the upper limit of 20. All model parameter estimates and their RSEs are presented in Table 1.

**Table 1.** Model parameters of PKPD model for *in vitro* data

Parameter	Description	Value	RSE (%)	SIR value (95% CI)
*k*_g_ (h^−1^)	Bacterial growth rate constant	1.47	3.40	1.46 (1.36–1.55)
*B*_max_ [log_10_ (cfu/mL)]	Maximum bacterial carrying capacity	12.3	1.20	12.3 (12.0–12.6)
*k*_d_ (h^−1^)	Natural bacterial death rate constant (fixed)	0.179	—	—
*B*_0_ [log_10_ (cfu/mL)]	Initial bacterial load	6.01	1.10	6.01 (5.90–6.11)
*E*_max,LZD_ (h^−1^)	Maximum LZD-mediated killing rate constant	1.97	8.50	1.97 (1.77–2.22)
EC_50,LZD_ (mg/L)	LZD concentration needed to reach 50% of *E*_max,LZD_	0.560	3.80	0.562 (0.524–0.602)
*γ*_LZD_ (−)	Hill coefficient (steepness of the concentration–LZD effect curve)	2.19	15.0	2.19 (1.81–2.64)
*t*_50,eff,LZD_ (h)	Time at which 50% of *E*_max,LZD_ is attained	2.24	9.90	2.24 (1.86–2.69)
*γ*_eff,LZD_ (−)	Hill coefficient (steepness of the time–effect curve)	1.31	31.6	1.36 (0.86–2.02)
*E*_max,VAN_ (h^−1^)	Maximum VAN-mediated killing rate constant	1.74	3.00	1.74 (1.64–1.82)
EC_50,VAN,0_ (mg/L)	VAN concentration needed to reach 50% of *E*_max,VAN_ in absence of resistance	0.245	2.00	0.246 (0.237–0.255)
*γ*_VAN_ (unitless)	Hill coefficient (steepness of the concentration–VAN effect curve, fixed)	20.0	—	—
*k*_ON,VAN_ (h^−1^ × mg^−1^ × L)	Second-order rate constant for development of VAN adaptive resistance	0.0635	5.30	0.0636 (0.0568–0.0701)
Slp_AR,VAN_ (−)	Linear coefficient that controls the adaptive resistance effect on EC_50,VAN,0_	3.43	6.70	3.42 (2.99–3.79)
RUV_in vitro_ [log_10_ (cfu/mL)]	*In vitro* residual unexplained variability (additive model in log scale)	0.512	6.30	0.517 (0.464–0.574)

### Standardized mouse pneumonia model efficacy experiments

Bacterial growth was achieved in the standardized mouse pneumonia model, with an increase in bacterial load of more than 1 log_10_ cfu/total lung at 26 h after bacterial inoculation (24 h after vehicle administration). Drug-mediated bacterial killing was dose-dependent, as shown in Figure S1. The lowest dose levels (LZD, 0.5–2 mg/kg q8h; and VAN, 1 mg/kg q4h) showed limited effect, and more than 1 log_10_ cfu/total lung bacterial growth was observed, i.e. similar bacterial load as the vehicle group at 24 h after the start of treatment. The VAN single-dosing group (40 mg/kg) presented an early bactericidal effect, but regrowth was observed at 24 h after the start of treatment. Bacterial stasis was achieved at the end of the study after dosing 8 mg/kg q8h (LZD) or 5 mg/kg q4h (VAN). A bactericidal effect (between 1- and 2-log kill) was seen at the highest LZD (40 mg/kg, 30 mg/kg q8h) and VAN (10–30 mg/kg q4h) doses, reaching a maximum reduction in bacterial load of 1.9-log and 2-log for LZD and VAN, respectively.

### Translation of PKPD model parameters from *in vitro* to *in vivo*

#### Changes in bacterial growth dynamics

The initial graphical exploration of the control mice group confirmed that the bacterial growth dynamics *in vivo* were slower and presented an initial growth delay compared with those *in vitro*. The *B*_max_ parameter could not be identified from the *in vivo* data because of sparse sampling, thus it was assumed *B*_max_ had been reached at 24 h and it was fixed to the median value of the maximum observed bacterial load in the vehicle group (7.8 log_10 _cfu/total lung). In order to account for the observed growth delay, a time-dependent sigmoidal function that modulated the growth rate parameter was included in the model. The time needed to reach half of the maximum growth rate was estimated to be 10.3 h (OFV dropped >40 points). The *in vivo* maximum growth rate parameter (0.85 h^−1^) was estimated to be 42% lower than the *in vitro* growth rate (1.47 h^−1^).

#### Adjustment of drug-mediated bacterial killing parameters

The bacterial killing parameters estimated from the *in vitro* data did not fit the observed data of the treated animals when only the bacterial growth dynamic parameters were adapted to *in vivo*. When the drug effect parameters were allowed to be re-estimated one by one, it was only the *Ratio* parameters for *E*_max_ (maximum bacterial killing rate constant) that resulted in a statistically significant improvement in the model fit. The estimates were 0.303 and 0.251 for LZD and VAN, respectively (OFV decreased 300.1 points). The maximum bacterial killing effect was consequently reduced by 69.7% and 74.9%, respectively.

**Figure 3.**
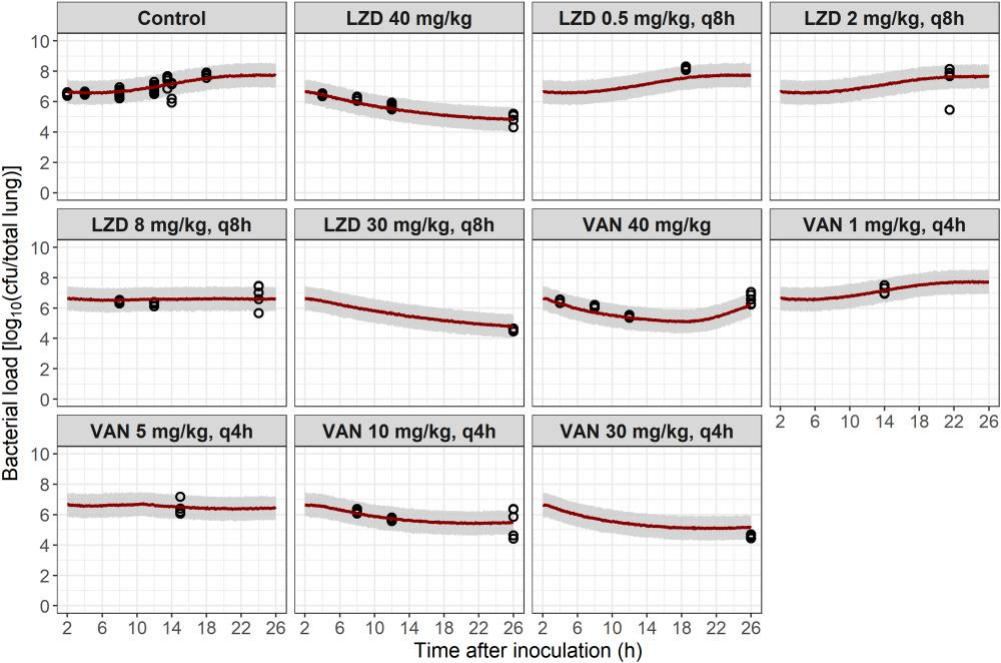
Bacterial load observed *in vivo* and simulations from the PKPD model for *in vivo* data considering model residual unexplained variability and parameter uncertainty. A total of 1000 studies with the same dosing regimens as the experimental data were simulated. LZD, linezolid. VAN, vancomycin. Open circles, observed bacterial load. Solid lines, median simulated bacterial load. Grey lines, 95% confidence interval for the simulated median.

An effect compartment model,^36^ describing a distribution delay to the site of action (lungs) from plasma, further reduced the OFV (147.9 points). The final PKPD model parameters for the *in vivo* data are presented in Table 2. Simulation-based plots displayed a good agreement between model simulations and observed *in vivo* data (Figure 3).

**Table 2.** Model parameters of PKPD model for *in vivo* data

Parameter	Description	Value	RSE (%)	SIR value (RSE%)
*k*_g,max_ (h^−1^)	Maximum bacterial growth rate constant	0.845	5.00	0.821 (0.782–0.862)
*B*_max_ [log_10_ (cfu/total lung)]	Maximum bacterial carrying capacity (fixed)	7.80	—	—
*k*_d_ (h^−1^)	Natural bacterial death rate constant (fixed)	0.179	—	—
*B*_0_ [log_10_ (cfu/total lung)]	Bacterial load at baseline (2 h after inoculation)	6.65	0.500	6.64 (6.54–6.74)
*t*_50,g_ (h)	Time at which 50% of *k*_g,max_ is attained	10.3	7.80	10.0 (8.96–11.1)
*γ*_g_ (−)	Hill coefficient (steepness of the time–growth curve)	1.53	10.6	1.54 (1.10–2.00)
Ratio_EMAX,LZD_ (−)	Change in *E*_max,LZD_ from the *in vitro* parameter	0.303	6.40	0.299 (0.269–0.330)
Ratio_EMAX,VAN_ (−)	Change in *E*_max,VAN_ from the *in vitro* parameter	0.251	6.40	0.249 (0.219–0.279)
*k*_e0,LZD_ (h^−1^)	LZD equilibration rate constant from plasma to effect compartment	0.0129	29.5	0.0130 (0.00915–0.0181)
*k*_e0,VAN_ (h^−1^)	VAN equilibration rate constant from plasma to effect compartment	0.0318	6.10	0.0318 (0.0285–0.0352)
RUV_in vivo_ [log_10_ (cfu/total lung)]	*In vivo* residual unexplained variability (additive model in log scale)	0.404	10.7	0.409 (0.365–0.459)

### Clinical simulations

**Figure 4.**
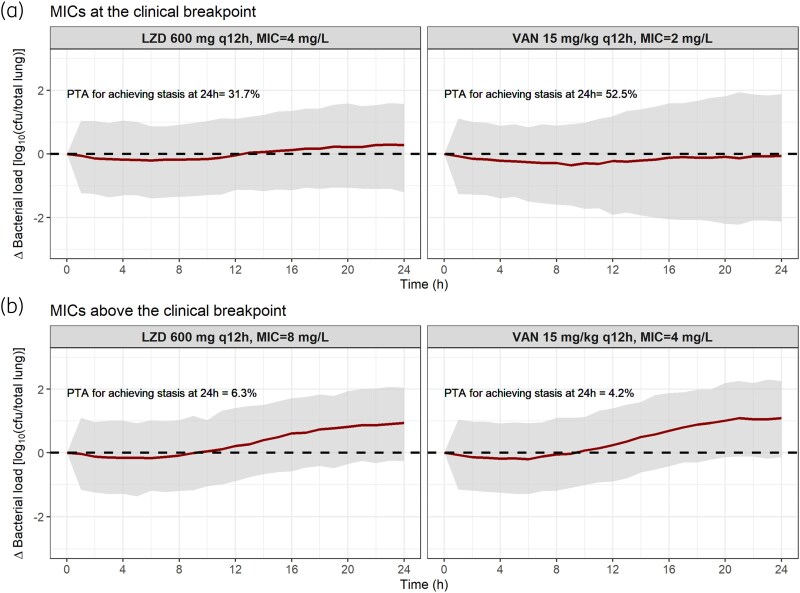
Simulated change in bacterial load from baseline over time for a virtual clinical population. A total of 1000 patients with standard dosing regimens for both LZD and VAN were simulated. Minimum inhibitory concentrations (MICs) defined as susceptible (a) or resistant (b) were included in the simulations. LZD, linezolid; VAN, vancomycin; PTA, probability of target attainment for achieving bacterial stasis. Solid lines, median simulated bacterial load. Grey areas, 95% confidence interval of the simulated median. Dashed line, bacteriostasis.

Changes in the bacterial load from baseline over time in a clinical population after the administration of either LZD or VAN for both susceptible and resistant MICs are displayed in Figure 4. Only 4.4% (VAN) and 6.1% (LZD) of the virtual patients with MICs above the breakpoint (LZD, 8 mg/L; VAN, 4 mg/L) achieved no net bacterial growth compared with the initial bacterial load (at least bacteriostasis) at 24 h, with a simulated median bacterial load of 1-log growth at that time. However, 35.4% (LZD) and 46.1% (VAN) of the simulated patients with MICs at the breakpoint (LZD, 4 mg/L; VAN, 2 mg/L) presented at least bacteriostasis, and the simulated median bacterial load was close to bacteriostasis.

## Discussion

In this work, we built a computational PKPD model, initially based on *in vitro* data, and thereafter updated by including *in vivo* data from a standardized mouse model for pneumonia. The use of longitudinal data allows us to capture mechanisms that otherwise would not be visible with single-timepoint observations. VAN showed bacterial regrowth *in vitro* at the last time points when exposed to concentrations close to the MIC, most likely because resistance developed to the drug over time. This was included in the PKPD model as an adaptive resistance term, which is a mechanism earlier described for VAN-mediated bacterial resistance for both MSSA^17^ and MRSA,^37^ as well as for other species such as *Escherichia coli*.^38^ The estimated bacterial growth rate constant (*k*_g_) for *in vitro* data was 1.47 h^−1^, corresponding to a doubling time of 0.47 h. Another model for MSSA by Wicha *et al*. estimated a similar value (1.56 h^−1^). The *k_g_* from a previous model for MRSA bacteria by Schmidt *et al*.,^39^ which was estimated to 1.19–1.21 h^−1^, was 18%–19% lower than our growth rate value. A similar LZD *EC_50_* parameter was found in the work by Wicha *et al*.^17^ (0.68 mg/L), but our parameter estimate was lower than the one by Schmidt *et al*.,^39^ being 1.39 mg/L (versus our value of 0.56 mg/L). However, our data could not be characterized by Wicha’s model and we developed our own model structure.

We also observed that 35.4–46.1% of the virtual clinical population with MICs lower than the clinical breakpoint (susceptible MICs) achieved bacteriostasis at 24 h after the administration of the standard-of-care dosing regimens for LZD and VAN, respectively. This result is in line with what is known for LZD, i.e. it exerts a bacteriostatic effect on most Gram-positive species, including *S. aureus*.^40^ On the other hand, VAN shows a bactericidal effect against susceptible *S. aureus* strains.^41^ However, our simulations demonstrated that a wide range of drug effect outcomes can be observed for the standard-of-care regimen due to therapeutic concentrations not being attained by all virtual patients. For example, it was predicted that 21.3% and 46.1% of patients with VAN MIC at the breakpoint (2 mg/L) would achieve at least 1-log bacterial killing or stasis, respectively, for a standard dosing regimen.

Data from different experiments were leveraged in steps to build the PKPD model. The initial base PKPD model was developed using *in vitro* data. This kind of experiment provides rich information about the bacterial growth dynamics, drug effect on bacterial killing, and potential drug resistance-mediated bacterial regrowth. However, the *in vitro* experiments cannot fully represent all the processes happening inside a living organism, both in terms of PKPD. The PKPD model for *in vitro* data was in the next step coupled with a mouse PK model to simulate potential outcomes from different dosing regimens to suggest suitable mouse study designs. Once the animal (sparser) data were available, we were able to test if the different features of the PKPD model were adequate for mouse data and we re-estimated those parameters differing significantly between *in vitro* and *in vivo*. We observed that the maximum drug-related bacterial killing effect rate constant (*E*_max_) *in vivo* (in lungs) was 69.7% (LZD) and 74.9% (VAN) lower than the same rate constant estimated when bacteria are exposed to the same drugs *in vitro*. Finally, from human PK analyses in the literature we could predict the drug concentrations over time to translate the final model to a human-like setting. These types of predictions can be of value in suggesting dosing regimens and scenarios of most interest to investigate in clinical trials or in clinical practice.

Animal data were obtained by successfully applying a standardized model for pneumonia in mice. This model had already been set up and tested for Gram-negative bacteria in an initiative to harmonize these types of experiments.^7^ We could here demonstrate that this model is also suitable for Gram-positive infections, achieving expected outcomes with the control compounds LZD and VAN. The standardization of experimental models allows the reduction of variability by using the same conditions within and across other laboratories, which could better inform decisions during the development of new antimicrobial compounds.

There were also limitations concerning this work. First, this translational project was carried out only using a single MRSA strain. From a translational point of view, it was prioritized to use a strain that showed virulence both *in vitro* and *in vivo*. Besides, the selected strain, MRSA 160079, CC398, was considered to be a representative strain that can cause both community-acquired and hospital-acquired pneumonia.^42–44^ A second limitation was that *in vivo* data were sparse for most of the dosing groups, which made it difficult to re-estimate all model parameters (and with adequate precision) using *in vivo* data from the model developed on *in vitro* data. In this work, there was a consideration of the balance between the robustness of the data set and the ethical (and resource) consideration of using as few mice as possible to generate experimental data. Thus, in order to mitigate this issue, one group per compound during the multiple-dosing study contained additional mice that were sacrificed earlier (at 8 and 12 h) to show the bacterial load over time. A third limitation was the lack of PK observations in the current study, which led to the use of literature mouse PK models and assuming the selected literature models were relevant to our data. Moreover, these models only described plasma PK and not lung (e.g. epithelial lining fluid, ELF) concentrations. This fact could explain why the *in vivo* PKPD models required effect compartments to account for the delay in plasma concentrations reaching the site of action. Penetration ratio in ELF for LZD is reported to be 1.15 in neutropenic mice^45^ and 1.00 in humans.^46^ For VAN, the mouse and human penetration ratios in ELF are 0.70–0.80 and 0.50, respectively, but as the plasma protein bindings are 17%–25% (mouse) and 50% (human), the total AUC in ELF is similar to the free AUC in plasma.^47,48^ Thus, the decrease in the maximum drug effect parameter values for both drugs *in vivo* should not be caused by a lower drug exposure in ELF, but because of other unexplored factors such as drug concentrations not reaching the infected lung tissue.^49^

In conclusion, the antimicrobial effect of LZD and VAN was studied against an MRSA clinical isolate using a translational approach that comprised *in vitro* time–kill experiments and *in vivo* treatment studies in a mouse pneumonia model. The developed PKPD model for *in vitro* data could characterize *in vitro* observations for all scenarios and informed the design of animal studies by describing key features such as potential resistance to VAN effect. The *in vivo* studies gave good results in terms of virulence and expected killing by control compounds, enabling further refinement of the PKPD model. The bacterial growth rates and the maximum drug effect parameters *in vivo* were lower than *in vitro* estimates. Simulations from the final *in vivo* PKPD model with human PK showed that the standard dosing regimens are expected to exert a bacteriostatic effect for a large percentage of the virtual population with LZD and vancomycin MICs equal to their respective breakpoints.

## Supplementary Material

dkaf140_Supplementary_Data
