# Supplementary material for: Model-based translation of the PKPD-relationship for linezolid and vancomycin on methicillin-resistant Staphylococcus aureus: from in vitro time–kill experiments to a mouse pneumonia model
Source: J Antimicrob Chemother. 2025 May 9;80(7):1860–8. doi: 10.1093/jac/dkaf140 (PMC12209834; doi:10.1093/jac/dkaf140)
Supplement: dkaf140_Supplementary_Data [file dkaf140_supplementary_data.docx]

**Supplementary material**

**Model-based translation of the PKPD-relationship for linezolid and vancomycin on Gram-positive bacteria: From in vitro time-kill experiments to a mouse pneumonia model**

Diego VERA-YUNCA^1^, Carina Sofia MATIAS^2^, Carina VINGSBO LUNDBERG^2^, Lena E. FRIBERG^1^

^1^ Department of Pharmacy, Uppsala University, Uppsala, Sweden

^2^ Antimicrobial Resistance and Pre-clinical Research - Bacteria, Parasites & Fungi, Statens Serum Institut, Copenhagen, Denmark

1. Experimental methods for *in vivo* treatment studies

Female BALB/c mice 9-10 weeks old (Envigo, Netherlands) were rendered neutropenic with cyclophosphamide pre-treatment at day -4 (150 mg/kg) and -1 (100 mg/kg) before being inoculated intranasally with 6-7 log_10_ cfu in a 40 µL volume under anaesthesia. For each experiment, the size of the inoculum was verified. Mice were monitored for clinical well-being throughout the study and were euthanized at designated experimental time points or upon reaching the humane endpoint. Lungs were collected and stored at -80°C until processing. Once thawed, the lungs were homogenized in sterile 0.9% NaCl using steel beads and a Qiagen Tissuelyser II. The homogenates were then serially diluted tenfold in saline, and 20 µl aliquots were plated onto agar. Plates were incubated at 35°C in ambient air for 18–24 hours.

1. Dosing selection for *in vivo* mouse studies

Single dosing studies were performed using a dose of 40 mg/kg for either linezolid or vancomycin. This dose value was chosen based on previous experience in-house with these compounds. These single dosing experiments were used as a proof-of-concept for the following multiple dosing experiments. Based on this preliminary data and the existing in vitro PKPD model, we predicted the potential effect of different multiple dosing regimens and we selected a range of lower and higher doses to have different outcomes that better informed our future *in vivo* PKPD model. Linezolid dose levels, administered every 8 hours for a total duration of 24 hours after bacterial inoculation: 0.5 mg/kg, 2 mg/kg, 8 mg/kg, 30 mg/kg q8h. Vancomycin dose levels, administered every 8 hours for a total duration of 24 hours after inoculation: 1 mg/kg, 5 mg/kg, 10 mg/kg, 30 mg/kg.

1. PKPD model equations
2. Bacterial growth model

$\frac{dA}{dt}= k_{g}\times A- k_{AD}\times A- \left( k_{d}+ {Eff}_{drug} \right)\times A$ (Eq. 1)

$\frac{dD}{dt}= k_{AD}\times A- k_{d}\times D$ (Eq. 2)

$k_{AD}= \left( k_{g}- k_{d} \right) \times\frac{A+D}{B_{max}}$ (Eq. 3)

Where *A* is the bacterial subpopulation that is susceptible to drug-induced bacterial killing, *D* is the dormant bacterial subpopulation, *k_g_* is the bacterial growth rate constant, *k_AD_* is the transfer rate constant from state *A* to state *D* that is modulated by the total bacterial concentration with respect to a maximum bacterial carrying capacity (*B_max_*); *k_d_* is the natural death rate constant with any drug effect, and *Eff_drug_* is the drug-induced bacterial killing rate constant (see *Drug effect model* section).

1. Drug effect model

${Eff}_{drug}= E_{max} \times\frac{f{Conc}^{\gamma}}{{{EC}_{50}}^{\gamma}+ {fConc}^{\gamma}}$ (Eq. 4)

Where *fConc* is the free drug concentration, *E_max_* is the maximum drug-induced bacterial killing effect, *EC_50_* is the drug concentration eliciting 50% of the maximum drug effect and *γ* is the Hill coefficient that describes how steep the concentration-effect E_max_ curve is.

${Eff}_{drug}(t)= {Eff}_{drug}(0) \times\frac{t^{\gamma_{eff}}}{{t_{50,eff}}^{\gamma_{eff}}+ t^{\gamma_{eff}}}$ (Eq. 5)

Where *t* is time in hours, *t_50,eff_* is the time at which half of maximum drug effect is exerted, and *γ_eff_* is the Hill coefficient controlling the steepness of the time-effect curve.

1. Adaptive resistance model

$\frac{d{AR}_{off}}{dt}= - k_{on} \times fConc \times{AR}_{off}$ (Eq. 6)

$\frac{d{AR}_{on}}{dt}= k_{on} \times fConc \times{AR}_{off}$ (Eq. 7)

Where the “degree” of adaptive resistance is represented by two compartments, *AR_off_* and *AR_on_*, *k_on_* is the adaptive resistance rate constant from *AR_off_* to *AR_on_* in presence of drug concentrations. At the start of the experiment, the amounts in *AR_off_* and *AR_on_* are 1 and 0, respectively. When bacteria are in contact with drug concentrations, there is a transfer of amount from *AR_off_* to *AR_on_*. The increasing amount inside *AR_on_* drives the resistance effect on the *EC_50_* parameter by increasing it linearly (supplementary equation 8), thus a higher concentration is needed to reach half of the maximum drug-mediated bacterial killing (supplementary equation 9).

${AR}_{eff}=1+ {Slp}_{AR} \times{AR}_{on}$(Eq. 8)

${EC}_{50}= {EC}_{50, 0} \times{AR}_{eff}$ (Eq. 9)

Where *AR_eff_* is the adaptive resistance effect on *EC_50_*, *Slp_AR_* is the linear coefficient that controls the effect of AR_on_ amount on *EC_50_*, and *EC_50,0_* is the original parameter in absence of any adaptive resistance effect.

1. Adjusting PKPD model parameter from *in vitro* to *in vivo*

$\theta_{invivo}=\theta_{invitro} \times{Ratio}_{\theta}$(Eq. 10)

Where *θ_invivo_* is the mouse PKPD parameter value, *θ_invitro_* is the original PKPD parameter value estimated with *in vitro* data, and *Ratio_θ_* is the estimated change (increase or decrease) in the parameter value from *in vitro* to *in vivo*. All *Ratio_θ_* parameters were fixed to 1 at the start of the *in vivo* modelling process. In a workflow that was similar to performing a stepwise covariate modelling (SCM)^1^ run, *Ratio_θ_* parameters were unfixed one by one for the forward step. Those that decreased the objective function (OFV) by more than 3.84 points were considered to be statistically significant. In the backward step, the previously selected ratios were fixed back to 1 one by one and those that increased the OFV by 10.83 points were kept in the final model.

1. Clinical EC_50_ parameter based on MIC

${EC}_{50, Patient}= {EC}_{50} \times\frac{{MIC}_{Patient}}{{MIC}_{Ref}}$ (Eq. 11)

Where *EC_50, Patient_* is the *EC_50_* value for a given simulated patient, *MIC_Patient_* is the MIC value for that patient, and *MIC_Ref_* is the reference MIC that was used for the development of the PKPD model *in vitro* and *in vivo*.

1. Delay in growth observed *in vivo*

$k_{g}(t)= k_{g,max} \times\frac{t^{\gamma_{g}}}{{t_{50, g}}^{\gamma_{g}}+ t^{\gamma_{g}}}$ (Eq. 12)

Where *k_g_(t)* is the time-varying bacterial growth rate constant for *in vivo* data, *k_g,max_* is the maximum attainable growth rate constant, *t_50,g_* is the time at which 50% of the maximum growth rate is reached and *γ_g_* is the parameter controlling the relationship between growth rate and time.

1. Effect compartment model

$\frac{d{Conc}_{eff}}{dt}= k_{e0} \times\left( fConc - {Conc}_{eff} \right)$ (Eq. 13)

Where *k_e0_* is a first-order rate that accounts for the delay in concentration equilibration relative to the plasma compartment, *fConc* is the free plasma concentration and *Conc_eff_* is the concentration in the effect compartment.

1. Literature mouse PK models

The selected linezolid mouse PK model presented a two-compartment model with separate saturable (Michaelis-Menten) and linear clearance pathways.^2^ The lack of established vancomycin mouse PK models led to inferring a one-compartment PK model from a non-compartmental analysis^3^ by deriving PK parameters from the area under the curve and half-life, giving a total clearance of 0.79 L*h^-1^*kg^-1^ and an apparent volume of distribution of 0.92 L/kg. Subcutaneous absorption information was missing. Thus, it was assumed the absorption process from the subcutaneous depot to plasma was fast (absorption rate constant *k_a_* fixed to 10 h^-1^). Unbound plasma drug concentration was computed for both drugs, with unbound fraction values of 0.76^4^ and 0.83^5^ for linezolid and vancomycin, respectively. Individual mouse weights were not available, so the average value of 25g was used for this analysis.

1. References

1. Khandelwal A, Harling K, Jonsson EN, *et al.* A Fast Method for Testing Covariates in Population PK/PD Models. *AAPS J* 2011; **13**: 464.

2. Bigelow KM, Deitchman AN, Li SY, *et al.* Pharmacodynamic Correlates of Linezolid Activity and Toxicity in Murine Models of Tuberculosis. *J Infect Dis* 2021; **223**: 1855.

3. Kim HK, Choi S-M, Kang G, *et al.* Comparison of In Vivo Pharmacokinetics and Pharmacodynamics of Vancomycin Products Available in Korea. *Yonsei Med J* 2020; **61**: 301–9.

4. Ahmed H, Bergmann F, Zeitlinger M. Protein Binding in Translational Antimicrobial Development-Focus on Interspecies Differences. *Antibiotics* 2022; **11**: 923.

5. Sandberg A, Jensen KS, Baudoux P, *et al.* Intra- and extracellular activity of linezolid against Staphylococcus aureus in vivo and in vitro. *J Antimicrob Chemother* 2010; **65**: 962–73.


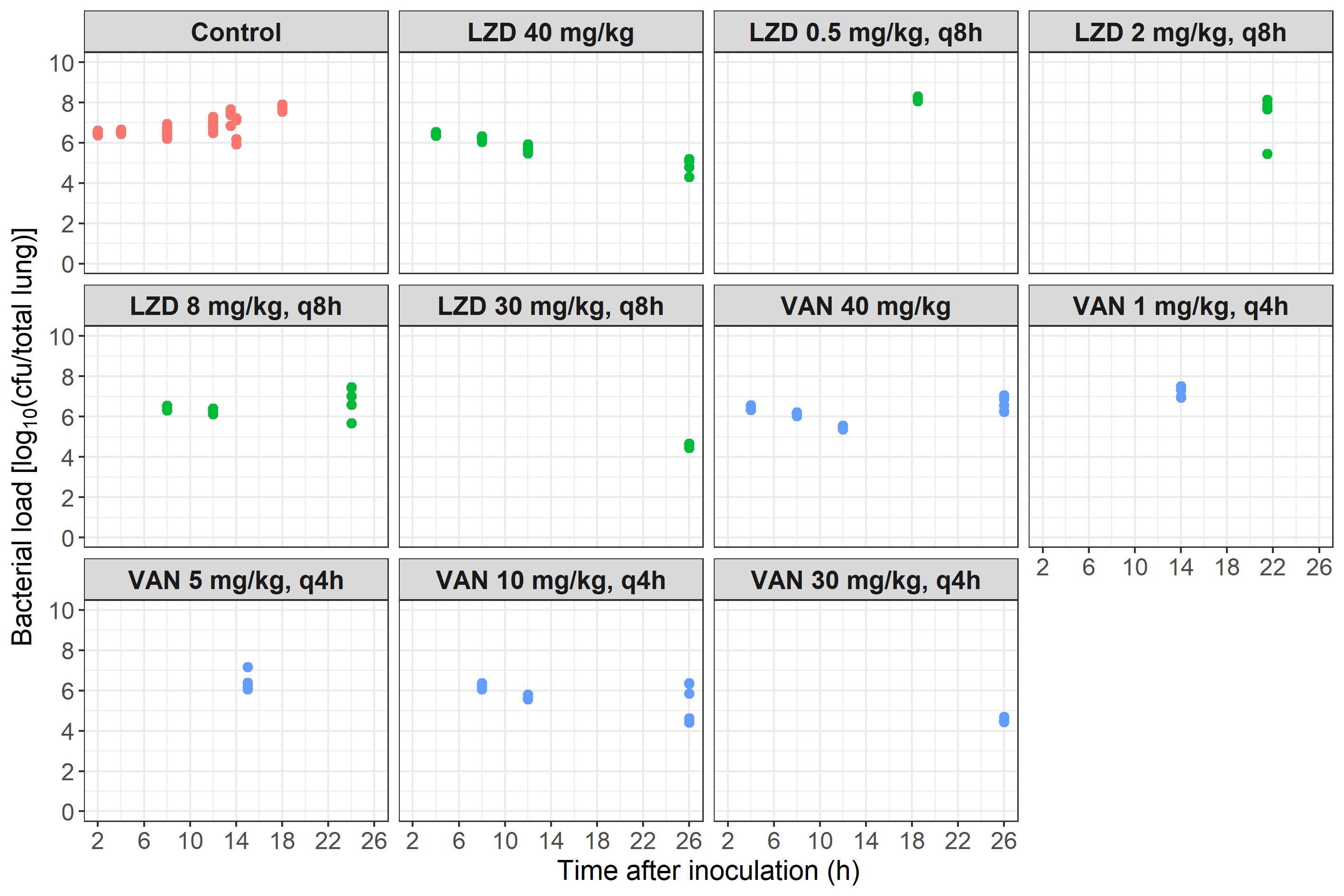


**Figure S1**. Bacterial load observed *in vivo* in control mice and mice treated with different dosing regimens of linezolid (LZD) or vancomycin (VAN).
